# Characterization of M-laurdan, a versatile probe to explore order in lipid membranes

**DOI:** 10.12688/f1000research.4805.2

**Published:** 2014-11-19

**Authors:** Serge Mazeres, Etienne Joly, Andre Lopez, Catherine Tardin

**Affiliations:** 1 CNRS, IPBS (Institut de Pharmacologie et de Biologie Structurale), Université de Toulouse, Toulouse, F-31077, France

## Abstract

Microdomains corresponding to localized partition of lipids between ordered and less ordered environments are the subject of intensive investigations, because of their putative participation in modulating cellular responses. One popular approach in the field consists in labelling membranes with solvatochromic fluorescent probes such as laurdan and C-laurdan. In this report, we describe a high-yield procedure for the synthesis of laurdan, C-laurdan and two new fluorophores, called MoC-laurdan and M-laurdan, as well as their extensive photophysical characterization. We find that the latter probe, M-laurdan, is particularly suited to discriminate lipid phases independently of the chemical nature of the lipids, as measured by both fluorescence Generalized Polarization (GP) and anisotropy in large unilamellar vesicles made of various lipid compositions. In addition, staining of live cells with M-laurdan shows a good stability over time without any apparent toxicity, as well as a wider distribution in the various cell compartments than the other probes.

## Introduction

Numerous physiological processes take place at the cell plasma membrane and its organization into domains participates in modulating many cellular responses ^1^. In animal cells, the external leaflet of plasma membranes (PMs) is mainly composed of phosphatidylcholine, sphingomyelin and cholesterol while the inner leaflet contains significant amounts of phosphatidylserine and phosphatidylethanolamine ^2^. Studies on *in vitro* model systems such as membranes isolated from cells or liposomes prepared either with synthetic lipids or extracted from PMs of cells from various sources have been instrumental in understanding the formation of lipid domains in biological membranes ^3, 4^. For example, pure sphingomyelin is known to form solid like phase (So) at physiological temperature ^5^, due to its long acyl chain. In cell membranes containing cholesterol, however, cholesterol and sphingomyelin have been shown to interact and form liquid ordered phases (Lo) surrounded by a liquid disordered phase (Ld) essentially comprised of phosphatidylcholine ^2^.

Results obtained with artificial model membranes are, however, difficult to transpose to natural membranes and research performed on intact live cells is preferred ^6^. In this context, fluorescent probes incorporated directly into cells are used extensively to reveal and characterize lipid domains, at sufficiently low concentrations that they should cause minimal disturbance to the membrane organization ^7, 8^.

For labelling biological membranes, two main classes of fluorescent molecules are usually used ^8^. The first one is composed of lipids such as phospholipids, sphingolipids, gangliosides or cholesterol that are attached to classical fluorescent dyes like fluorescein isothiocyanate (FITC) and nitro benzoxadiazol (NBD) ^9, 10^. Such probes can be used to image domains because of their preferential partitioning to certain phases in models as well as in cell membranes ^11^. The phase preference of these dyes is a subtle equilibrium between the fluorescent part of the molecule, the acyl chains and the interaction with the surrounding lipids of the membrane, thus some narrow changes in molecular interactions may shift the probe partitioning ^12, 13^. The second class of dyes developed for studying order in biological membranes are molecules with fluorescent moieties which are highly sensitive to the surrounding micro-environment. One of the most commonly used families of probes is laurdan and its derivatives ^14, 15^, whilst other probes include Nile Red ^16^, di-n-ANEPPDHQ ^17^, 3-hydroxy-flavone ^18^ and 2-anthroyl lipid derivatives ^19^. For such probes no preferential phase partitioning is expected.

Laurdan, and other related fluorescent molecules containing 2-hydroxy-6-dodecanoyl naphthalene are environment-sensitive dyes which exhibit a large Stokes shift correlated to the polarity of the surrounding medium ^20, 21^. This effect comes from intramolecular charge transfer (ICT) when solvent relaxation occurs, as well as from local excitation (LE), without solvent relaxation ^22^. In polar solvents, the emission is red shifted compared to apolar ones ^23^. When laurdan is embedded in lipid bilayers that contain no sterol, a transition from solid (So) to liquid disorder (Ld) phases results in a large red shift ^20^. The fluorescence modulation can originate from complex processes including reduced solvent relaxation due to lipid packing and high micro-viscosity, abrupt water molecules gradient through lipid bilayers ^24^, possible H-bonding with donor lipid groups ^25^ and/or dyes bending and sliding along the z-axis ^26^. Complex lipid mixtures including sphingomyelin and cholesterol can also modulate the fluorescence parameters due to specific lipid/lipid and lipid/dye interactions ^27^. Recently, Kim *et al.* ^15^ synthesized and characterized C-laurdan, which also shows great sensitivity to changes in lipid order in the bilayers, but labels the plasma membrane of cells more efficiently than laurdan. Because C-laurdan is not commercially available, we decided to synthesize it ourselves. To achieve this, we revisited the synthesis of 2-hydroxy-6-dodecanoyl naphthalene, the precursor of laurdan, which also provided us with access to two new candidate probes molecules that we called M-laurdan and MoC-laurdan, in addition to laurdan and C-laurdan. Here we report the thorough characterization of the photophysical properties of these four probes in parallel, both in solvents and in model membranes. We also show their possible use to label live tissue culture. We show that, among the four candidate probes, M-laurdan looks as a particularly interesting alternative to laurdan or C-laurdan to label live cells.

## Materials and methods

### Chemicals and solvents

Chemicals and solvents were all purchased from Sigma-Aldrich and ultra-pure water was used for buffer preparation (Milli-Q 18 MΩ, Millipore, France).

### Dioxane/water mixtures and pure solvents

A series of media covering a range of dielectric constants from 2 to 60, corresponding to the variation of the polarity of a bilayer from the center to the lipid head group, was obtained by mixing the right amount of dioxane in water. The dielectric constant was taken as the linear combination of a mixture of non-polar (1,4-dioxane, ε _20°C_=2.2) and polar (Water, ε _20°C_=80.1) solvents according to their molar fraction ^28^. Other experiments were carried out using pure solvents with dielectric constant ranging from 4.8 to 33 and classified as non-polar (chloroform), polar aprotic (dichloromethane, acetone, acetonitrile, dimethylformamide) and polar protic (ethanol, methanol) media. For all experiments, the dyes were dissolved in solvents to a final concentration of 1 µM, starting from dried residues.

### Large Unilamellar Vesicles (LUVs)

DPPC (1,2-dipalmitoyl-sn-glycero-3-phosphocholine), DOPC (1,2-dioleoyl-sn-glycero-3-phosphocholine), POPC (1-palmitoyl-2-oleoyl-sn-glycero-3-phosphocholine) and cholesterol were purchased from Sigma-Aldrich. Porcine brain sphingomyelin (BSM, containing 50% C _18:0_ and 21% C _24:1_ as major compounds) and egg sphingomyelin (PSM containing 86% C _16:0_ as major compound) were from Avanti Polar Lipids. Large Unilamellar Vesicles (LUVs) were obtained by sonication. The amounts of chloroform stocks of lipids and dyes, calculated to obtain a final concentration of 100 µM for the lipids and 1 µM for the probes, were mixed in glass tubes. The chloroform solvent was first evaporated under nitrogen flow and then under vacuum for 2 hours. 3 ml of MOPS buffer (3-(N-morpholino) propane sulfonic acid, 10 mM, pH 7.3, NaCl 100 mM, EDTA 10 µM) was then added (at a temperature 5°C above the gel-to-liquid phase transition *T*_m_ for DPPC, BSM and PSM, and at room temperature for the other lipids and lipid mixtures). The tubes were then sonicated for 5 minutes (immersion tip diameter 3 mm, delivered power 18 W, Bioblock, France) and kept at 4°C overnight before use. Liposomes obtained by sonication are usually mostly comprised of SUVs (15 – 50 nm in diameter), but the size of those quickly increases due to events of fusion, resulting in a population of larger vesicles comprising a mixture of relaxed SUVs, MLVs and OLVs. Characterization of those lipid vesicles by dynamic light scattering (DLS) showed that the main population of vesicles had sizes ranging from 150 to 350 nm in diameter depending on the mix of lipids used to prepare the liposomes. For simplicity, we simply refer to such vesicles as LUVs in the rest of the manuscript. The OD at 500 nm was checked systematically, to ensure that it remained below 0.05 to avoid any inner filter effect.

The compositions of the lipid mixtures were chosen on the basis of the literature (see Table S1).

### Fluorescence spectroscopy

Absorption spectra were recorded on a Specord 205 spectrophotometer (Analytik Jena, Germany), fluorescence spectra on a FLSP920, lifetimes on a TCSPC Model 199 (both from Edinburgh Instruments, UK) and anisotropy on an automated home-built set-up allowing to control and change the temperature of the sample.

Fluorescence emission spectra were recorded over the range from 370 to 600 nm with excitation set to 360 nm on a thermostatic sample holder. Lifetimes were recorded at 460 nm, with 380 nm pulsed LED for excitation (PLS 370, Picoquant, Germany). POPOP was used as a reference for quantum yield (Φ=0.97 in cyclohexane) and lifetime (τ=1.35 ns in ethanol) ^29^. Anisotropy was recorded by integrating the fluorescence emission through a band pass filter (450/50) and polarizers with appropriate orientations (excitation set to 360 nm) at temperatures ranging from 10 to 60°C (2°C step increments).

Quantum yield was calculated using the following equation ^29^:

$$\phi =\frac{S}{S_{\text{r}}}\frac{{\text{OD}}_{\text{r}}}{\text{OD}}{\left(\frac{\text{n}}{{\text{n}}_{\text{r}}}\right)}^{2}\Phi_{\text{r}}$$

where S is the area of the integrated intensity, OD is the optical density and n is the refractive index (r for reference sample).

Fluorescence decay times were analyzed as a sum of exponentials using a least-squares algorithm according to the following equation ^30^:

$$\text{I(t)}=\sum_{\text{i=1}}^{\text{n}}\frac{{\text{I}}_{\text{i}}}{\tau_{\text{i}}}\exp^{-\frac{\text{t}}{\tau_{\text{i}}}}$$

in which I _i_ is the steady-state intensity and τ _i_ is the lifetime ( $\frac{{\text{I}}_{\text{i}}}{\tau_{\text{i}}}=\alpha_{1}$).

Mean fluorescence lifetime was calculated as follows ^31^:

$$\langle \tau \rangle =\frac{\sum_{\text{i}}\alpha_{\text{i}}\tau_{\text{i}}^{\text{2}}}{\sum_{\text{i}}\alpha_{\text{i}}\tau_{\text{i}}}$$

were is α _i_ the normalized pre-exponential factor and τ _i_ is the lifetime of the decay i.

The reported anisotropies were corrected for instrument response (G-factor) and calculated using the following expression ^32^:

$$\langle \text{r}\rangle =\frac{{\text{I}}_{\text{vv}}-{\text{GI}}_{\text{vh}}}{{\text{I}}_{\text{vv}}+2{\text{GI}}_{\text{vh}}}\;\;\;\;\;\;\;\;\;\text{G}=\frac{{\text{I}}_{\text{hv}}}{{\text{I}}_{\text{hh}}}$$

were I _xy_ corresponds to the fluorescence intensity recorded for vertical (v) or horizontal (h) polarizer position (x for excitation path, y for emission path).

The Generalized Polarization (GP) was calculated from the measured fluorescence intensities at 440 and 490 nm ^33^:

$$\text{GP}=\frac{{\text{I}}_{440}-{\text{I}}_{490}}{{\text{I}}_{440}+{\text{I}}_{490}}$$

### Labelling of tissue culture cells

COS7 cells, obtained from ATCC, were grown in DMEM + 10% fetal calf serum and passaged 1/10 by trypsinization every three or four days. For microscopy, COS7 cells were left to adhere overnight on sterile glass coverslips (Thermo Scientific Menzel). Cells were then rinsed twice with PBS with no serum, before incubating at 37°C with the probes diluted to 10 μM in PBS with no serum, for 15 minutes for C-laurdan and 30 minutes for the other three. The cells were then rinsed twice with PBS + 10% FCS warmed to 37°C.

### Two-photon fluorescence microscopy

Cell imaging was performed on a LSM 710 NLO-Meta confocal microscope with spectral detection (Zeiss, Germany) coupled to a two-photon laser source (Chameleon Vision II, Coherent, France). Images were taken through a 40x/1.2W objective under controlled environment (37°C, 5% CO _2_). Images were recorded in one pass from 420 to 600 nm with 10 nm steps (excitation, 720 nm). GP maps were calculated with ImageJ (NIH) from images recorded at 440 and 490 nm using the formula described above and a custom-built macro (available upon request).

## Results and discussion

### Synthesis

To simplify and ameliorate the synthesis of the laurdan family dyes, we revisited the synthesis procedure of their precursor, 2-hydroxy-6-dodecanoyl naphthalene, by taking advantage of a Fries rearrangement in methane sulfonic acid, as proposed by Commarieu *et al.* ^34^. Using commercially available standard reactants, naphthol-2 and lauroyl chloride, this precursor was produced with a yield of nearly 100% in two reaction steps including the Fries rearrangement.

**Figure 1.**
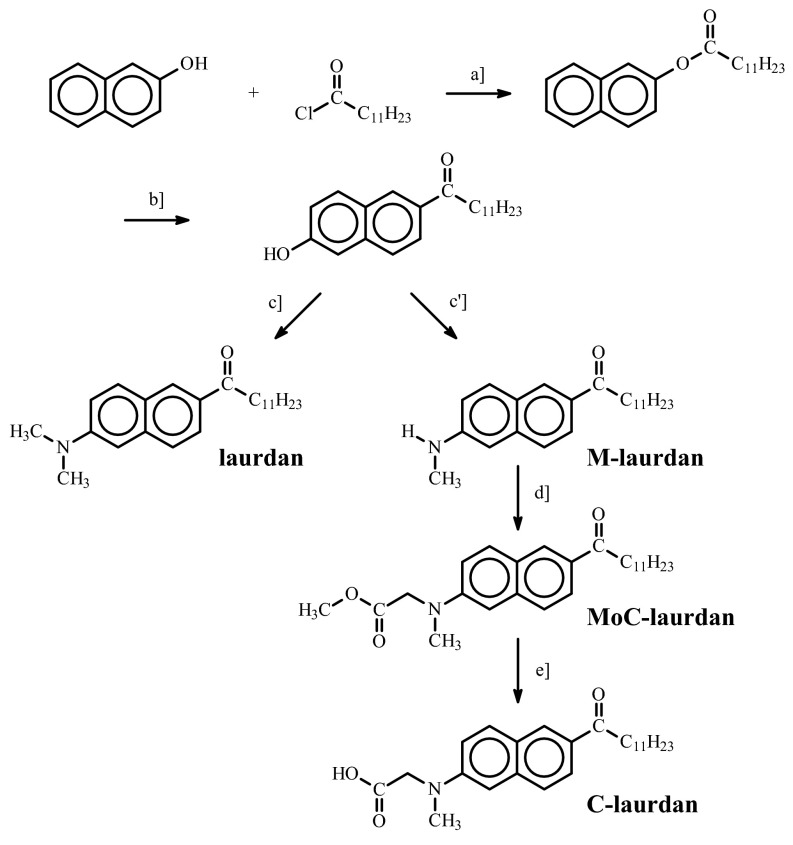
Synthesis pathway of laurdan, M-laurdan, MoC-laurdan and C-laurdan. Reagents and conditions: **a**] Naphthol-2, Lauroyl chloride (triethanolamine); **b**] Fries rearrangement, Methane sulfonic acid; **c**] (CH _3_)2NH HCl, Na _2_S _2_O _5_, NaOH, H _2_O; **c'**] CH _3_NH _2_ HCl, Na _2_S _2_O _5_, NaOH, H _2_O; **d**] BrCH _2_COOCH _3_, Na _2_HPO _4_, CH _3_CN; **e**] KOH, EtOH.

Based on this common precursor, four fluorescent probes related to laurdan were obtained: 6-dodecanoyl-2-(dimethylamino) naphthalene (laurdan), 6-dodecanoyl-2-(methylamino) naphthalene (M-laurdan), 6-dodecanoyl-2-[N-methyl-N-(methoxycarbonyl) amino] naphthalene (MoC-laurdan) and 6-dodecanoyl-2-[N-methyl-N-(carboxy-methyl) amino] naphthalene (C-laurdan) ( Figure 1). Laurdan was produced using dimethylamine. M-laurdan, MoC-laurdan and C-laurdan were produced following the protocol reported by Kim *et al.* ^15^. All dyes were produced with satisfactory yields using conventional chemistry facilities. Finally, the four compounds were purified by thin layer chromatography and characterized by ^1^H-NMR (see SI attribution peaks). Dyes formulae, molecular weight and basic photophysic characteristics are presented in Table 1.

**Table 1.** Dyes formulae, molecular weight and basic photophysic characteristics measured in chloroform.

Name	Laurdan	M-laurdan	MoC-laurdan	C-laurdan
Formula	C _24_H _35_NO 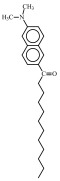	C _23_H _33_NO 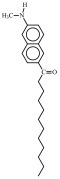	C _26_H _37_NO _3_ 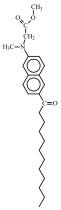	C _25_H _35_NO _3_ 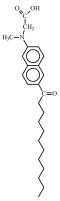
M.W.(g/mol)*	353.55	339.52	411.59	397.56
Molar extinction coefficient ε (M ^-1^cm ^-1^)**	19500	14100	11100	12200
Quantum yield Φ***	0.61	0.58	0.48	0.56
Brightness (ε-Φ in CHCl _3_)	11900	8200	5300	6800

*Calculated using C=12.011, H=1.008, N=14.007 and O=15.999 (atomic weights). **ε measured at 360 nm in CHCl _3_. ***Φ measured in CHCl _3_.

### Photophysical characteristics in solvents

**Figure 2.**
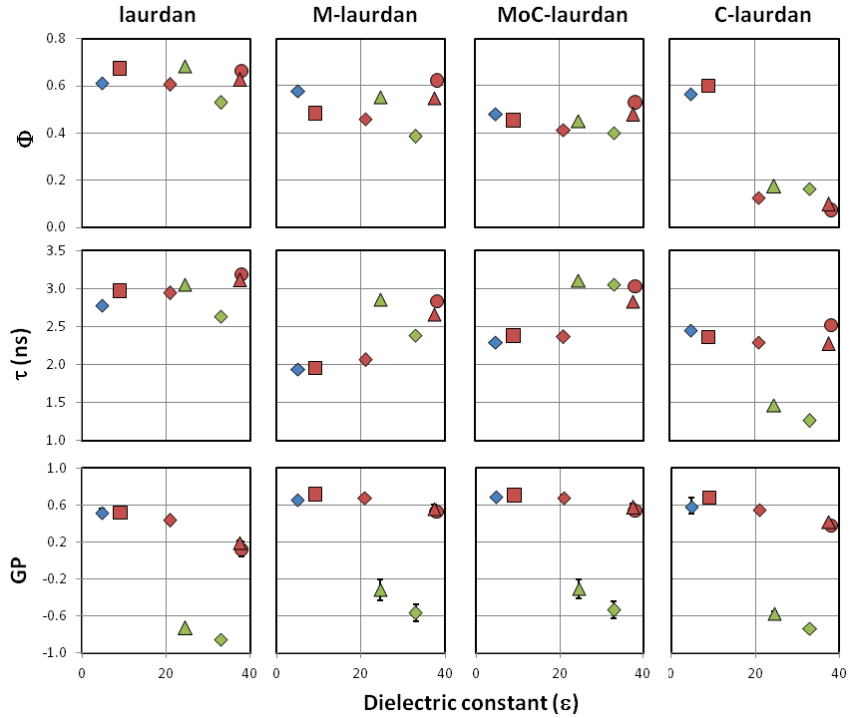
Spectroscopic properties (quantum yield, Φ; lifetime, τ; Generalized Polarization, GP) of laurdan, M-laurdan, MoC-laurdan and C-laurdan in various solvents. Blue, non-polar solvent ( ◆ chloroform), red, polar aprotic solvents ( ■ dichloromethane, ◆ acetone, ▲ acetonitrile, ● dimethylformamide), green, polar protic solvents ( ▲ ethanol, ◆ methanol). Experiments were performed twice and reported values are mean ± SD (not visible error bars are below the size of symbols).

**Supplemental Figure S1.**
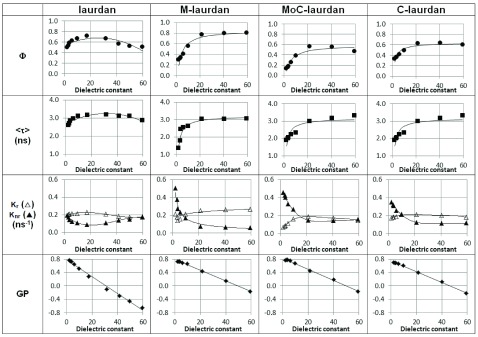
Photophysical characteristics in dioxane/water mixtures. Spectroscopic properties of the four probes in dioxane/water mixtures covering a dielectric constant range from 2 to 60. Quantum yields and mean lifetimes were measured. Deexcitation constants k _r_ (Δ), k _nr_ (▲) and Generalized Polarization GP were calculated.

In order to evaluate the possible influence of the nature of the solvent beyond its dielectric constant on the photophysical properties of the four probes, we carried out measurements in dioxane/water mixtures with dielectric constants spanning a broad range of values ( Figure S1), and next in a variety of pure solvents with dielectric constants ranging from 4.8 to 33 ( Figure 2 and Table 2). From the steady-state measurements in dioxane/water mixtures, for all four dyes, we found a linear correlation between the measured GP and the dielectric constant of the media ( Figure S1). In contrast with these results obtained in dioxane/water mixtures, low values of GP were seen only in the pure solvents ethanol and methanol, which are both polar protic solvents with dielectric constants above 20 (ε _EtOH_=25 and ε _MeOH_=33). The other photophysical characteristics, quantum yield and lifetime (Φ, τ), were found to be rather constant for all the probes except for C-laurdan, for which the quantum yield was seen to decrease very significantly in protic solvents and polar aprotic solvents with ε>20.

**Table 2.** Photophysical parameters of the dyes dissolved in pure solvents at a final concentration of 1 µM. ε refers to the solvent dielectric constant, quantum yields ϕ are measured using POPOP as reference (ϕPOPOP=0.97 in cyclohexane), lifetimes τ are recorded at 460 nm (excitation, 380 nm) and reported along with the goodness of the fit χ ^2^ (POPOP was used as reference for deconvolution, τPOPOP=1.35 ns in ethanol), kr (ϕ/τ) and knr ((1-ϕ)/τ) are the deexcitation constants deduced from quantum yields and lifetimes, Generalized Polarization, GP, are calculated from emission intensities at 440 and 490 nm.

Solvent			laurdan									MoC-laurdan							
	ε		ϕ		χ²	τ (ns)	kr (ns ^-1^)	knr (ns ^-1^)		GP		ϕ		χ²	τ (ns)	kr (ns ^-1^)	knr (ns ^-1^)		GP
**Nonpolar**																			
CHCl _3_	4.8		0.61		1.53	2.78	0.22	0.14		0.48		0.48		3.03	2.30	0.21	0.23		0.72
**Polar aprotic**																			
DCM	9.1		0.67		1.99	2.97	0.23	0.11		0.48		0.45		4.62	2.38	0.19	0.23		0.73
Acetone	21.0		0.61		1.53	2.94	0.21	0.13		0.40		0.41		1.81	2.36	0.17	0.25		0.71
MeCN	37.5		0.63		1.82	3.11	0.20	0.12		0.16		0.48		1.50	2.83	0.17	0.18		0.61
DMF	38.0		0.67		1.81	3.19	0.21	0.10		0.04		0.53		1.46	3.03	0.17	0.16		0.54
**Polar protic**																			
EtOH	24.6		0.68		1.81	3.05	0.22	0.10		-0.72		0.45		1.77	3.10	0.15	0.18		-0.20
MeOH	33.0		0.53		1.80	2.64	0.20	0.18		-0.85		0.40		1.93	3.05	0.13	0.20		-0.44

Solvent			M-laurdan									C-laurdan							
	ε		ϕ		χ²	τ (ns)	kr (ns ^-1^)	knr (ns ^-1^)		GP		ϕ		χ²	τ (ns)	kr (ns ^-1^)	knr (ns ^-1^)		GP
**Nonpolar**																			
CHCl _3_	4.8		0.58		6.74	1.95	0.30	0.22		0.68		0.56		2.50	2.46	0.23	0.18		0.67
**Polar aprotic**																			
DCM	9.1		0.48		2.70	1.95	0.25	0.27		0.74		0.60		1.87	2.36	0.25	0.17		0.72
Acetone	21.0		0.46		4.99	2.06	0.22	0.26		0.70		0.13		1.87	2.29	0.05	0.38		0.57
MeCN	37.5		0.55		1.63	2.66	0.21	0.17		0.60		0.10		2.43	2.27	0.04	0.40		0.43
DMF	38.0		0.62		1.68	2.83	0.22	0.13		0.51		0.07		2.31	2.52	0.03	0.37		0.32
**Polar protic**																			
EtOH	24.6		0.55		2.48	2.85	0.19	0.16		-0.20		0.17		2.72	1.46	0.12	0.57		-0.61
MeOH	33.0		0.38		2.38	2.38	0.16	0.26		-0.48		0.16		4.45	1.26	0.13	0.67		-0.74

**Supplemental Figure S2.**
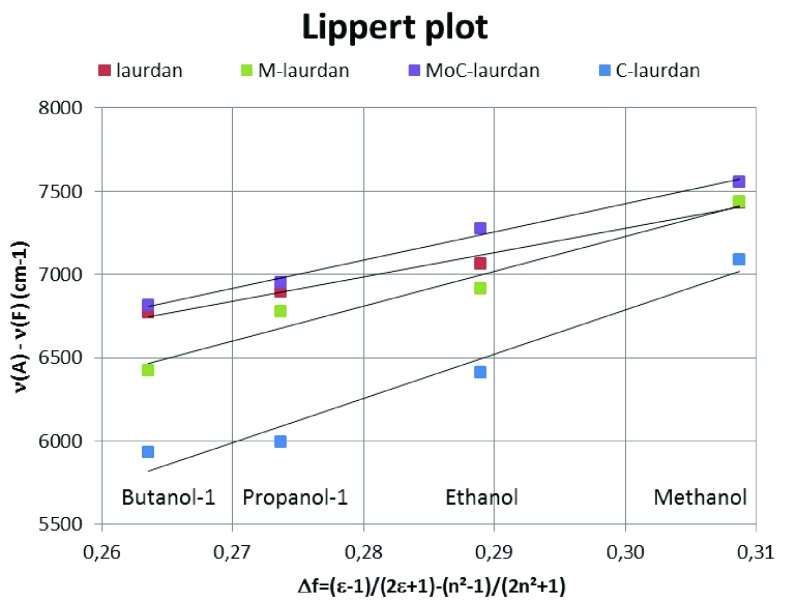
We built the Lippert plots of laurdan, M-laurdan, MoC-laurdan and C-laurdan in alcohols (butanol-1, propanol-1, ethanol and methanol). The Lipper plots shows the Stokes shift ( $\bar{\nu_{A}}$– $\bar{\nu_{F}}$) versus the orientation polarizability (Δf) and therefore gives an estimation of the fluorophores solvent sensitivity. The linearity of the laurdan family curves can be interpreted as a general solvent effect in the spectral shift. Besides, the similarity in the slopes obtained for the different curves drives us to conclude that the different dyes are similarly sensitive to solvent polarity.

For all four probes, the GP was low only in protic solvents, i.e. ethanol and methanol ( Figure 2), presumably because of those solvents’ capacity to make hydrogen bonds. The Lippert plots of the four probes were found to be linear with a similar slope, which leads us to conclude that the different dyes are similarly sensitive to solvent polarity ( Figure S2).

**Supplemental Figure S3.**
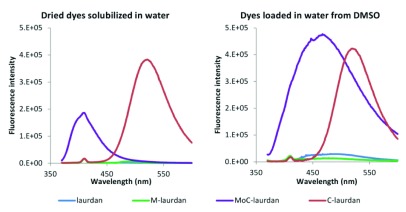
Fluorescence emission in water. Left panel: For all four dyes, the volumes of chloroform stock solutions corresponding to 3 nanomoles were dried in test tubes under nitrogen, followed by further evaporation with a vacuum pump for 2 hours. Water (3 ml) was added to each sample (final theoretical concentration: 1 µM), which were vortexed for 30 seconds at room temperature and emission spectra were then measured with a FLSP920 (Edinburgh Instruments, Scotland) fluorimeter with excitation set to 360 nm. Right panel: Dyes were dissolved in DMSO to obtain stock solutions of 1 mM. 3 microliters from those DMSO stock solutions were injected in 3 ml of water (final concentration of 1 µM). Emission spectra were then measured with a FLSP920 (Edinburgh Instruments, Scotland) fluorimeter. The very small peak at 410 nm corresponds to the Raman of water, as a consequence of the excitation set to 360 nm.

Next, we characterized the fluorescence of the four dyes in water at a final concentration of 1 µM. For this, we used two procedures of solubilization: either directly from dried dye residues, or indirectly, using dimethyl sulfoxide (DMSO) loading ( Figure S3). For the indirect procedure, similarly to what is commonly performed for labelling cells, dye stock solutions were prepared at 1 mM in DMSO and injected at 1:1000 into water ^7^. For laurdan and M-laurdan, no fluorescence and extremely low absorption were detected in the solution obtained directly from dried residues. In the solutions obtained by DMSO loading, very weak fluorescence signals were detected, which may correspond either to slightly improved solubility or to the probes remaining in complexes with DMSO. Our data are in agreement with the well-known insolubility of Laurdan in water ^35^. The fluorescence intensity of the MoC-laurdan obtained from dried residue was significant but with a very marked blue shift, suggesting that dyes were not surrounded by water molecules and quite possibly aggregated. For the MoC-laurdan solution obtained by DMSO loading, the fluorescence was very high, with the emission maximum near 460 nm at the time of the measure. This suggested that the probe remained in an environment with a lower dielectric constant than pure water and thus probably remained conjugated to some DMSO molecules for extended periods of time. For C-laurdan, Kim *et al.* ^15^ have previously reported that this dye is soluble in water. Accordingly, we detected an intense fluorescence, with the spectra being independent of the solubilization procedure utilized. The emission spectrum was red-shifted, with a maximum at 520 nm compared to 425 nm in chloroform, which could correspond to the dye sensing a protic surrounding.

Altogether, we conclude that i) laurdan and M-laurdan are probably almost insoluble in water when starting from dried residues, and have very low fluorescence in water when injected with DMSO, ii) MoC-laurdan can fluoresce in pure water, but may either aggregate if placed in water directly, or remain associated to DMSO when this is used to dissolve the probe iii) C-laurdan is soluble and fluoresces in pure water.

### Insertion of the fluorophores into LUVs

For labelling model bilayers, we employed the most adequate procedure which consists in the direct assembly of fluorescently labelled LUVs. This entails direct mixing of lipids and fluorescent probes in chloroform, followed by steps of desiccation to remove the organic solvent, and then by rehydration in buffer, which includes a sonication step (see Materials and methods). In contrast, this direct procedure cannot be applied to label live cell membranes. An indirect procedure is then applied where the fluorescent solvatochromic lipid probes are commonly solubilized into DMSO before large dilution in serum-free culture medium and addition to cells ^36^.

**Supplemental Figure S4.**
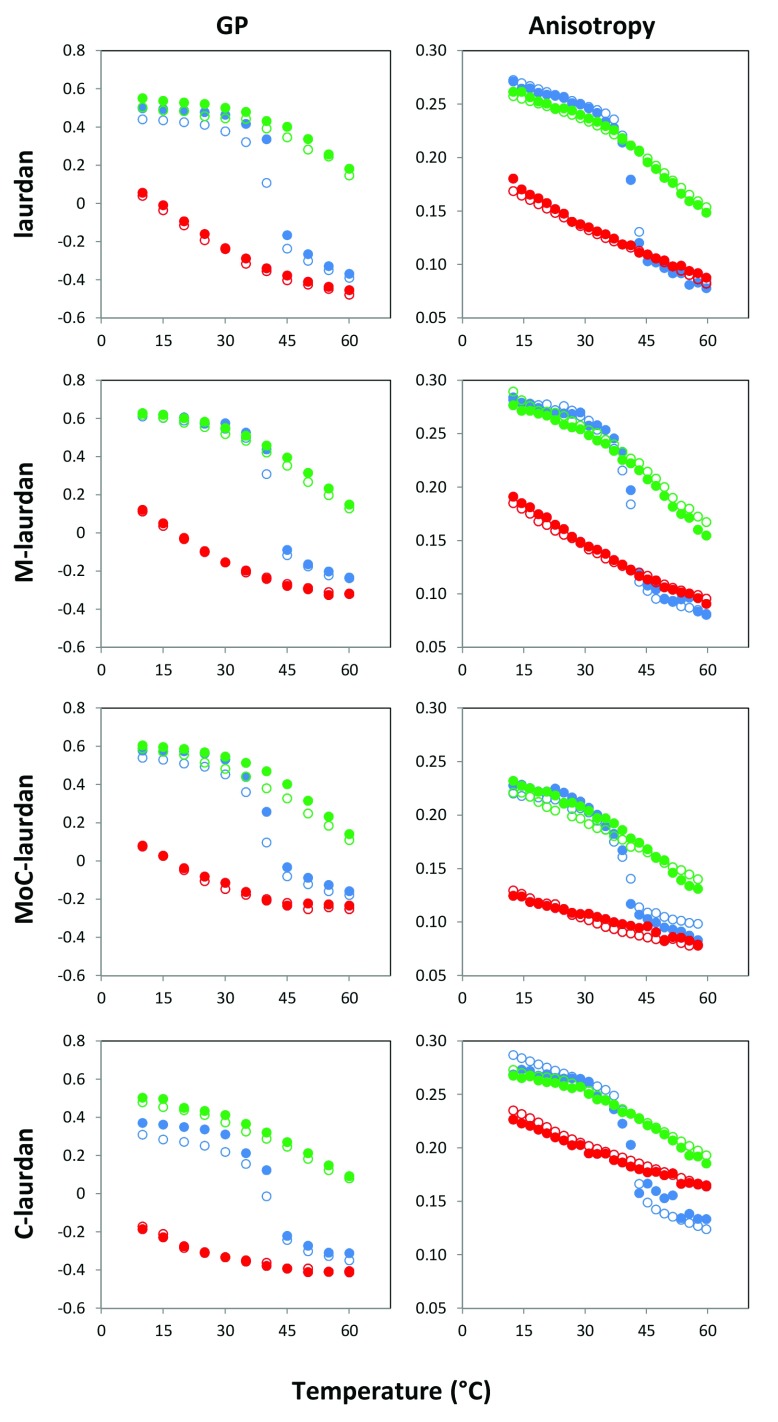
Comparison of GP and anisotropy experiments on LUVS labeled either by direct mixing of lipids and dyes or by the DMSO loading method. LUVs made of DPPC, DPPC/chol (6:4) and POPC (100 µM, sample volume 3 ml) were either prepared in presence of each of the four dyes laurdan, M-laurdan, MoC-laurdan and C-laurdan, or unlabeled LUVs were loaded with the dyes dissolved in DMSO. The final dye concentrations were 1 µM and the DMSO/water ratio 1:1000. GP and anisotropy were recorded as a function of temperature. Symbols: blue for DPPC, green for DPPC/chol (6:4) and red for POPC, filled symbols for LUVs prepared in presence of dyes and open symbol for DMSO loading method. As can be seen from the graphs, the two procedures used to label liposomes with dyes gave extremely similar results.

Using laurdan, M-laurdan, MoC-laurdan and C-laurdan on LUVs made of DPPC, DPPC/cholesterol (6:4) and POPC, we performed GP and anisotropy measurements to compare both types of samples and we found no detectable difference, suggesting that the presence of low concentrations of DMSO in the samples does not have any significant effect on this kind of probes ( Figure S4).

**Figure 3.**
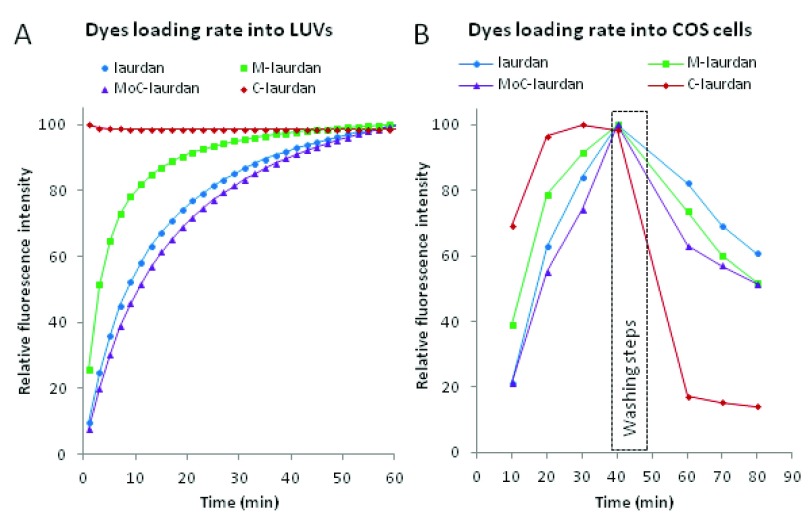
Time dependence of dyes incorporation into LUVs and COS7 cells. **A** (Left panel): Incorporation of the dyes into LUVs resulted in an increase of the fluorescence intensity over time. Unlabeled LUVs made of DPPC/chol (6:4) (100 µM, sample volume 3 ml) were mixed with dyes dissolved in DMSO. The final dye concentrations were 1 µM and the DMSO/water ratio 1:1000. Measurements were carried out at 20°C and fluorescence intensities were recorded at 440 nm. The fitting curve was obtained using a one site binding equation. **B** (Right panel): For flow cytometry (FACS), cells were harvested as a single cell suspension in tissue culture medium + serum after trypsinization and washed twice with PBS with no serum. For each probe, 1 µl of stock solution at 2 mM in DMSO was mixed with 2 million cells in 200 µl PBS (10 µM final) at room temperature. After 10 minutes, the tubes were placed in a water bath at 37°C for 30 min, before cells were washed twice in 5 ml and resuspended in a final volume of 1 ml with PBS + 10% serum warmed to 37°C. Cells were then further incubated for 20 min at 37°C. Every 10 minutes throughout the procedure, the volume corresponding to 250.000 cells was taken from each sample, placed on ice in a final volume of 500 µl ice-cold PBS + 10% serum, and submitted to FACS analysis on a LSR II flow cytometer with a UV laser and using the channel with a 450/50 BP filter.

Next, we examined the capacity of the four probes to label LUVs, using the common procedure of adding them solubilized in DMSO as it is the case for live cell membrane labelling. We first estimated the time required for the insertion of 50% of the fluorophores from the measurement of fluorescence intensity over time after injection of dyes on LUVs made of DPPC/cholesterol (6:4) ( Figure 3A-left panel). For laurdan, M-laurdan and MoC-laurdan, fluorescence was found to increase over time, to reach a plateau around 60 minutes, with the first fluorescence measurement recorded 1 minute after injection being close to zero. From our data, we estimated the half insertion times to be in the range of three to 6 minutes for M-laurdan, and 10 to 15 minutes for laurdan and MoC-laurdan. In contrast, C-laurdan fluorescence intensity recorded 1 minute after injection was already high and stayed constant during the course of the experiment. For MoC-laurdan, the maximum value of the emission spectra shifted from 460 to 425 nm over the course of the experiment, suggesting that the probe was still mostly conjugated with DMSO at the early time points (< 5 minutes) and inserted progressively in the bilayer at later times. The large differences observed between the insertion times of the various probes probably originate from differences in their hydrophilic head volumes. C-laurdan is the only probe among the four to carry a head group which can be partially ionized, i.e. the carboxylic group. This probably contributes greatly to C-laurdan's water solubility and fast membrane incorporation. All in all, however, the insertion times of all four probes are compatible with membrane labelling of live cells.

### Probing model bilayers in pure Ld states

**Figure 4.**
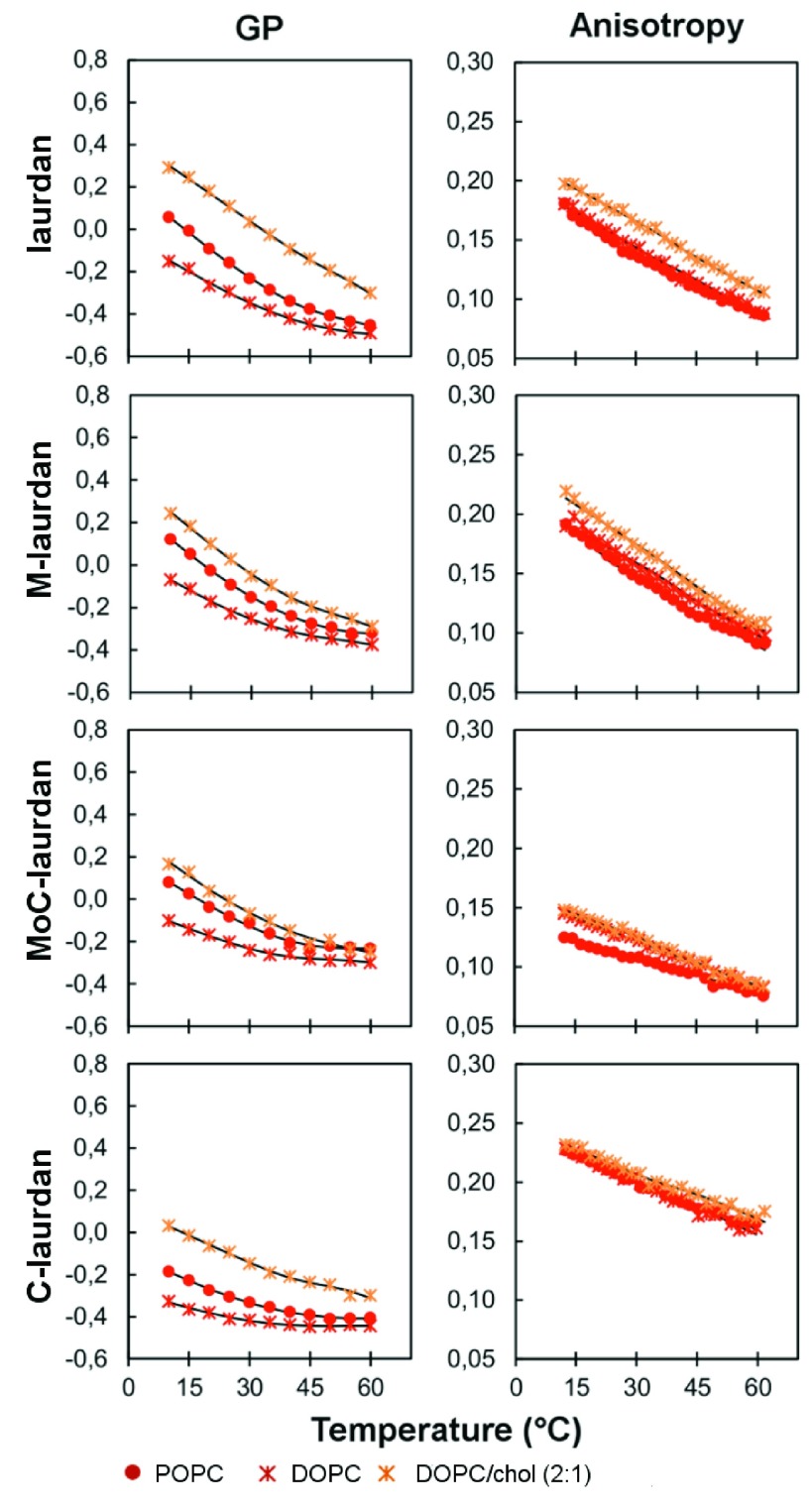
GP and anisotropy of laurdan, M-laurdan, MoC-laurdan and C-laurdan dyes in model membrane in Ld phase (orange/red color codes depicting liquid disordered phases).

To record the bilayer characteristics measured with the four probes we chose two phospholipids: POPC, a major component of biological membranes as well as DOPC, a phospholipid seldom encountered in nature, but which is broadly used as a model to generate raft-like domains in membranes ^37^. At room temperature, pure POPC and pure DOPC are known to assemble into Ld phases ^38, 39^. Accordingly, GP values, as a function of temperature, remained low for all probes, in agreement with the high level of hydration of the lipid bilayer in Ld states ( Figure 4). GP measured on pure DOPC were systematically below those measured in pure POPC, indicating a higher degree of hydration in DOPC bilayers.

When anisotropy was measured, as a function of temperature, laurdan and M-laurdan gave similar values, whereas lower values were found for MoC-laurdan and higher for C-laurdan ( Figure 4). This could stem from a difference in the transverse positioning of the dye into the bilayer affecting their rotational mobility, or from different fluorescence lifetimes.

The fluorescence lifetimes were thus measured and found to be very similar to one another for the first three: laurdan (<τ> _POPC_=3.19 ns, <τ> _DOPC_=3.04 ns at 20°C), M-laurdan (<τ> _POPC_=3.16 ns, <τ> _DOPC_=3.33 ns at 20°C) and MoC-laurdan (<τ> _POPC_=3.03 ns, <τ> _DOPC_=3.17 ns at 20°C). For C-laurdan, fluorescence lifetimes were 30% shorter compared to the others (<τ> _POPC_=2.26 ns, <τ> _DOPC_=2.13 ns at 20°C) ( Table 3). The rotational relaxation time is directly correlated with the order parameter through the Perrin-Weber equation. Those were thus calculated from anisotropies and lifetimes values ( Table S2). Laurdan, M-laurdan and C-laurdan exhibit similar values indicating that those dyes have similar constraints in the bilayers. For MoC-laurdan, the lower rotational relaxation time could thus indicate a more superficial insertion of the probe that may be due to its bulky head group.

**Table 3.** Lifetimes measured for dyes in various lipid environments at 20°C and 50°C (excitation 380 nm, lifetimes recorded at 460 nm, POPOP used as reference, τ=1.35 ns in ethanol). In the table, the lines in blue correspond to bilayers in So phase, in green in Lo, and in red in Ld.

Sample	laurdan												
	20°C							50°C					
	χ²	α1 (%)	τ1 (ns)	α2 (%)	τ2 (ns)	<τ>		χ²	α1 (%)	τ1 (ns)	α2 (%)	τ2 (ns)	<τ>
DPPC	4.43	56	6.18	44	0.53	**5.82**		4.26	100	2.89	-	-	**2.89**
PSM	1.29	57	4.96	43	2.50	**4.28**		1.84	78	3.15	22	1.06	**2.97**
BSM	1.55	67	4.25	33	1.96	**3.82**		2.15	68	2.79	32	1.03	**2.53**
DPPC/chol (6:4)	4.53	60	5.75	40	0.61	**5.41**		2.03	100	4.03	-	-	**4.03**
PSM/chol (2:1)	3.31	69	5.58	31	0.61	**5.34**		1.56	100	3.68	-	-	**3.68**
BSM/chol (2:1)	1.50	90	4.73	10	2.52	**4.61**		1.57	100	3.20	-	-	**3.20**
POPC/chol (2:1)	1.45	100	4.35	-	-	**4.35**		1.89	100	2.79	-	-	**2.79**
DOPC/BSM/chol (1:1:1)	1.49	94	4.42	6	2.05	**4.36**		1.63	38	3.09	62	2.92	**2.99**
DOPC/PSM/chol (1:1:1)	1.73	81	4.43	19	0.70	**4.30**		1.62	100	2.88	-	-	**2.88**
DOPC/chol (2:1)	1.49	100	3.75	-	-	**3.75**		1.66	100	2.48	-	-	**2.48**
DOPC	1.50	79	3.23	21	1.44	**3.04**		1.84	65	2.81	35	0.83	**2.54**
POPC	2.69	79	3.28	21	0.43	**3.19**		4.04	100	2.49	-	-	**2.49**

Sample	M-laurdan												
	20°C							50°C					
	χ²	α1 (%)	τ1 (ns)	α2 (%)	τ2 (ns)	<τ>		χ²	α1 (%)	τ1 (ns)	α2 (%)	τ2 (ns)	<τ>
DPPC	1.75	81	5.05	19	1.05	**4.86**		1.31	100	3.28	-	-	**3.28**
PSM	1.36	58	4.69	42	2.14	**4.06**		1.64	82	3.23	18	1.67	**3.07**
BSM	3.11	28	5.09	72	2.97	**3.81**		3.07	83	2.82	17	1.86	**2.70**
DPPC/chol (6:4)	5.17	60	4.80	40	0.67	**4.45**		1.42	100	3.83	-	-	**3.83**
PSM/chol (2:1)	2.66	68	4.55	32	0.51	**4.36**		1.67	100	3.67	-	-	**3.67**
BSM/chol (2:1)	1.29	61	4.83	39	2.90	**4.29**		1.43	100	3.31	-	-	**3.31**
POPC/chol (2:1)	1.50	100	3.98	-	-	**3.98**		2.12	100	3.06	-	-	**3.06**
DOPC/BSM/chol (1:1:1)	1.43	61	4.19	39	3.83	**4.06**		1.65	100	3.29	-	-	**3.29**
DOPC/PSM/chol (1:1:1)	2.50	82	4.06	18	2.12	**3.85**		1.63	100	3.15	-	-	**3.15**
DOPC/chol (2:1)	1.49	100	3.81	-	-	**3.81**		1.41	59	3.33	41	2.78	**3.12**
DOPC	1.76	92	3.40	8	1.89	**3.33**		1.41	65	3.37	35	2.01	**3.04**
POPC	5.99	64	3.42	36	0.64	**3.16**		1.64	75	3.11	25	1.14	**2.90**

Sample	MoC-laurdan												
	20°C							50°C					
	χ²	α1 (%)	τ1 (ns)	α2 (%)	τ2 (ns)	<τ>		χ²	α1 (%)	τ1 (ns)	α2 (%)	τ2 (ns)	<τ>
DPPC	1.49	85	5.46	15	2.29	**5.24**		1.38	18	4.89	82	2.61	**3.28**
PSM	1.50	42	3.46	58	1.82	**2.77**		1.72	68	3.00	32	1.06	**2.73**
BSM	1.84	18	3.29	82	1.22	**2.00**		2.54	34	2.44	66	0.87	**1.80**
DPPC/chol (6:4)	1.37	85	4.91	15	1.62	**4.73**		1.38	29	4.29	71	2.79	**3.38**
PSM/chol (2:1)	2.25	43	5.09	57	1.90	**4.03**		1.56	38	4.10	62	1.81	**3.14**
BSM/chol (2:1)	1.33	41	4.12	59	2.04	**3.26**		1.54	47	3.36	53	1.43	**2.73**
POPC/chol (2:1)	1.59	80	3.39	20	1.22	**3.21**		2.01	60	2.96	40	1.54	**2.60**
DOPC/BSM/chol (1:1:1)	1.37	54	3.74	46	1.78	**3.17**		1.75	51	3.18	49	1.19	**2.66**
DOPC/PSM/chol (1:1:1)	2.12	62	3.59	38	1.50	**3.16**		1.77	53	3.26	47	1.93	**2.81**
DOPC/chol (2:1)	1.55	73	3.41	27	1.89	**3.15**		1.95	72	3.04	28	1.69	**2.80**
DOPC	1.53	71	3.47	29	1.44	**3.17**		1.70	79	3.25	21	1.50	**3.06**
POPC	1.88	82	3.19	18	1.56	**3.03**		2.04	70	3.02	30	1.32	**2.76**

Sample	C-laurdan												
	20°C							50°C					
	χ²	α1 (%)	τ1 (ns)	α2 (%)	τ2 (ns)	<τ>		χ²	α1 (%)	τ1 (ns)	α2 (%)	τ2 (ns)	<τ>
DPPC	1.34	94	5.08	6	1.35	**5.02**		2.39	26	2.90	74	1.34	**2.01**
PSM	1.48	42	3.64	58	1.59	**2.87**		1.83	24	2.80	76	1.20	**1.87**
BSM	1.62	42	3.77	58	1.61	**2.96**		2.35	35	2.46	65	0.93	**1.83**
DPPC/chol (6:4)	1.66	85	4.83	15	1.12	**4.69**		1.67	73	3.07	27	2.33	**2.91**
PSM/chol (2:1)	4.04	59	5.39	41	0.61	**5.05**		1.67	100	3.22	-	-	**3.22**
BSM/chol (2:1)	1.34	83	4.65	17	2.49	**4.44**		1.47	75	2.77	25	1.39	**2.57**
POPC/chol (2:1)	1.63	90	3.62	10	1.66	**3.53**		1.75	35	2.62	65	1.44	**2.02**
DOPC/BSM/chol (1:1:1)	1.65	62	3.81	38	1.90	**3.36**		1.50	17	3.34	83	1.59	**2.12**
DOPC/PSM/chol (1:1:1)	2.51	58	4.30	42	2.01	**3.72**		2.15	46	2.65	54	1.27	**2.16**
DOPC/chol (2:1)	1.50	60	3.00	40	1.27	**2.62**		1.99	22	2.53	78	1.16	**1.69**
DOPC	2.30	47	2.60	53	1.34	**2.13**		2.35	16	2.95	84	1.01	**1.70**
POPC	2.17	63	2.54	37	1.48	**2.26**		2.70	16	2.65	84	1.04	**1.56**

In addition to these bilayers comprised of single components, we also carried out experiments on a lipid bilayer composed of two components, which has been previously characterized as being in Ld phase at room temperature: DOPC and cholesterol with a 2:1 molar ratio ^27, 40, 41^. Whilst only few differences were observed on the anisotropy measurements, the GP values were clearly higher than on pure DOPC, in agreement with the well-described capacity of cholesterol to expel water molecules from the bilayer interface ^42^.

### Probing model bilayers undergoing So to Ld transition

**Figure 5.**
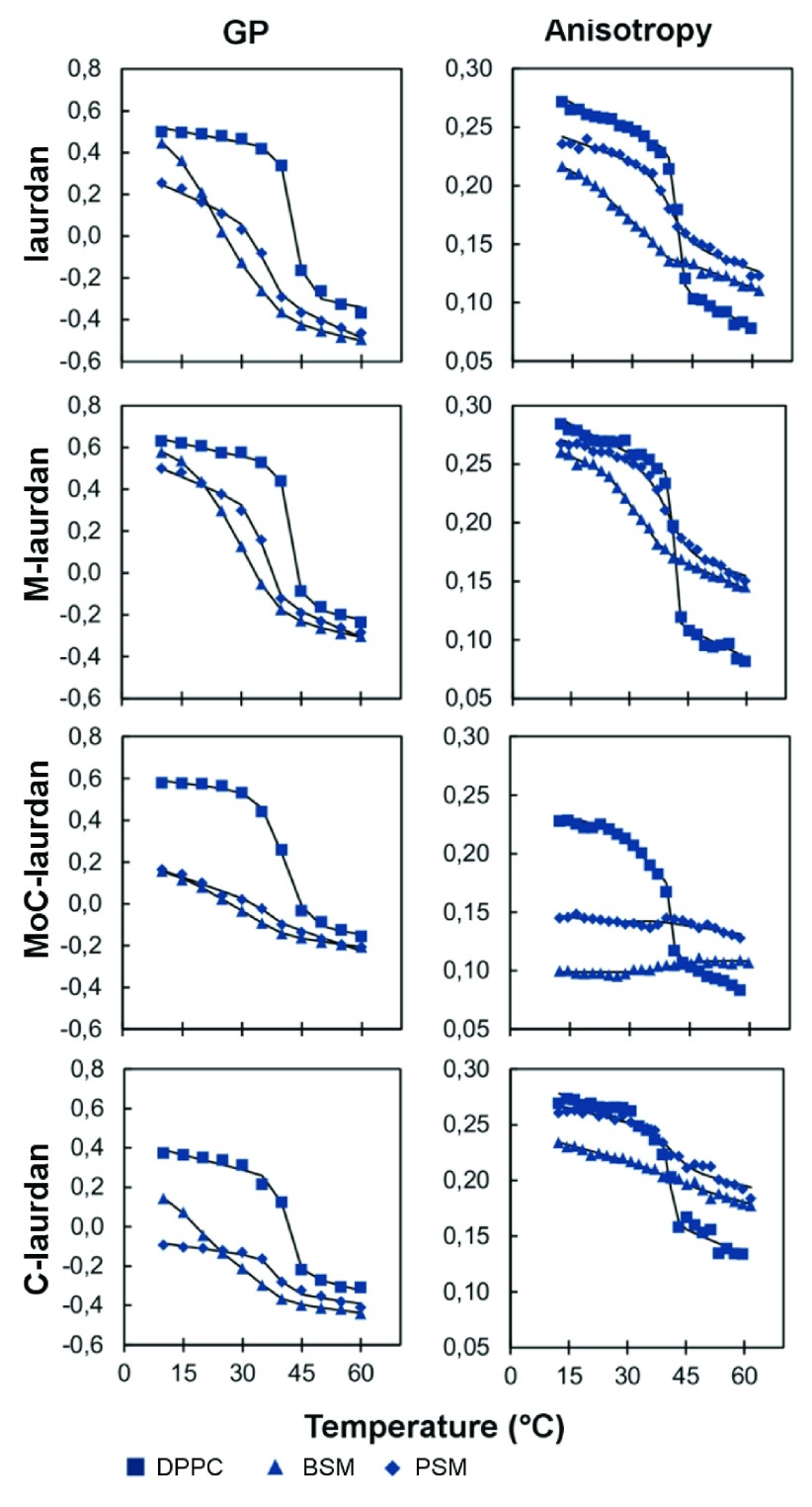
GP and anisotropy of laurdan, M-laurdan, MoC-laurdan and C-laurdan dyes in model membrane undergoing So to Ld transition (blue color code depicting solid ordered phases at low temperature).

We then turned our interest to the characterization of the four probes in lipid bilayers in solid-like phases ( Figure 5). For bilayers made of single components, we chose either pure DPPC, which is classically used for model bilayers, or sphingomyelins, as they are major components of the microdomains observed in cells’ plasma membranes. Two sources of sphingomyelins were used, which carry acyl chains with different lengths: BSM (mostly C _18:0_) and PSM (mostly C _16:0_). When incorporated into a DPPC bilayer, all four probes showed a steep decrease for both GP and anisotropy when heated above the melting transition of 41°C, indicating that the four probes are equivalently sensing the phase behavior from So to Ld occurring in the bilayer, in agreement with the results found in the literature for laurdan ^43^. At room temperature, i.e. in a solid DPPC bilayer, the lifetimes measured for the four probes were all much longer than the ones measured in the Ld phase, ranging from 4.86 to 5.82 ns ( Table 3).

**Supplemental Figure S5.**
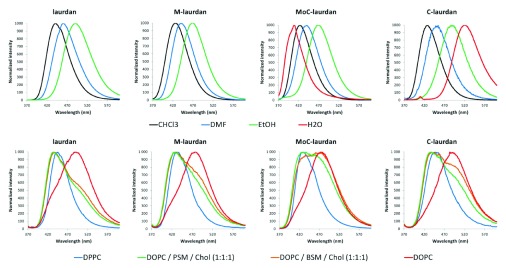
Normalized emission spectra of dyes in solvents and lipid vesicles for So, Lo and Ld phases. Upper panels show normalized emission spectra of laurdan, M-laurdan, MoC-laurdan and C-laurdan in chloroform, Dimethylformamide, ethanol and water (when detectable, i.e. only for Moc-laurdan and C-laurdan). The blue shift seen for the emission spectrum for MoC-laurdan in water is attributed to the fluorescence of non hydrated aggregates, whereas C-laurdan which is highly soluble in water undergoes the expected red shift. Lower panels show emission spectra of laurdan, M-laurdan, MoC-laurdan and C-laurdan in LUVs made of DPPC (So, blue), DOPC/PSM/chol 1:1:1 (Lo, green), DOPC/BSM/chol 1:1:1 (Lo, orange) and DOPC (Ld, red) at 25°C.

The same measurements performed on bilayers made of sphingomyelins gave results which proved more complicated to interpret. First, the fluorescence emission spectra measured in sphingomyelins below the melting transition showed a broader peak when compared to the spectrum obtained with DPPC ( Figure S5). As seen in Figure 5, laurdan and M-laurdan exhibited a steep fall in GP around 35°C for BSM ^44, 45^ and 41°C for PSM ^31^. By contrast, MoC-laurdan was quite insensitive to temperature transitions in both sphingomyelins, whilst C-laurdan showed a remarkable reduction of amplitude for the PSM transition. For anisotropy measurements, the steep decrease characteristic of the solid to liquid phase transition ^46^ was only seen for laurdan and M-laurdan, whilst MoC-laurdan did not show any significant change of anisotropy induced by the temperature, and C-laurdan only to a reduced extent.

The strong contrast between the results found with DPPC and sphingomyelins might be related to the differences in their backbone structure since both types of lipids carry phosphocholine as head groups: DPPC can only accept a single hydrogen bond while sphingomyelin possesses an additional hydrogen bond accepting group. Also, both types of sphingomyelins used here (BSM and PSM) are derived from natural sources. As such, they contain a variety of fatty acids of different chain lengths. This will greatly impact on their phase behavior, and can thus contribute to explain why the results of anisotropy measurements do not give results which are as clear-cut as with DPPC. The very weak correlation between anisotropy and temperature observed for MoC-laurdan may be a consequence of the very low values of its fluorescence lifetimes in sphingomyelins (<τ> _BSM_=2.00 ns, <τ> _PSM_=2.77 ns at 20°C) compared to DPPC (<τ> _DPPC_=5.24 ns at 20°C), which may in turn be due to a poor insertion of the dye into the lipid bilayer as confirmed by rotational relaxation time calculation ( Table S2).

In sphingomyelin bilayers, M-laurdan, laurdan and to a lesser extent C-laurdan exhibited the expected steep decrease in their GP, with temperature corresponding to a melting transition. The differences observed in the steepness of the curve at the melting transition, as well as in the width of the fluorescence emission spectrum, could also arise from a shallow insertion of the probes in the sphingomyelin membranes due to the repulsion between the amino groups on the sphingomyelins and the one carried by the fluorescent probes, as already proposed for laurdan ^46^. This effect would be reduced for M-laurdan because it has the smallest head group of the family. On the contrary, when the probes were inserted in pure BSM or PSM bilayers, it was only with laurdan and M-laurdan that the fluorescence decay exhibited a lifetime component close to 4 ns at 20°C ( Table 3). This longer lifetime, compared to MoC-laurdan and C-laurdan (values less than 3 ns), might explain the better capacity of laurdan and M-laurdan to reveal phase transitions in sphingomyelin bilayers. In fact, the longer lifetimes of M-laurdan and laurdan when those are inserted into bilayers in So phase (between 3.81 and 5.82 ns) may allow anisotropy measurements to provide information about the order parameter of the membrane, since it would be long enough to unravel the frozen state of So phase, as does the diphenylhexatriene DPH with a fluorescence lifetime around 8–10 ns ^47^.

### Probing model bilayers in Lo states

**Figure 6.**
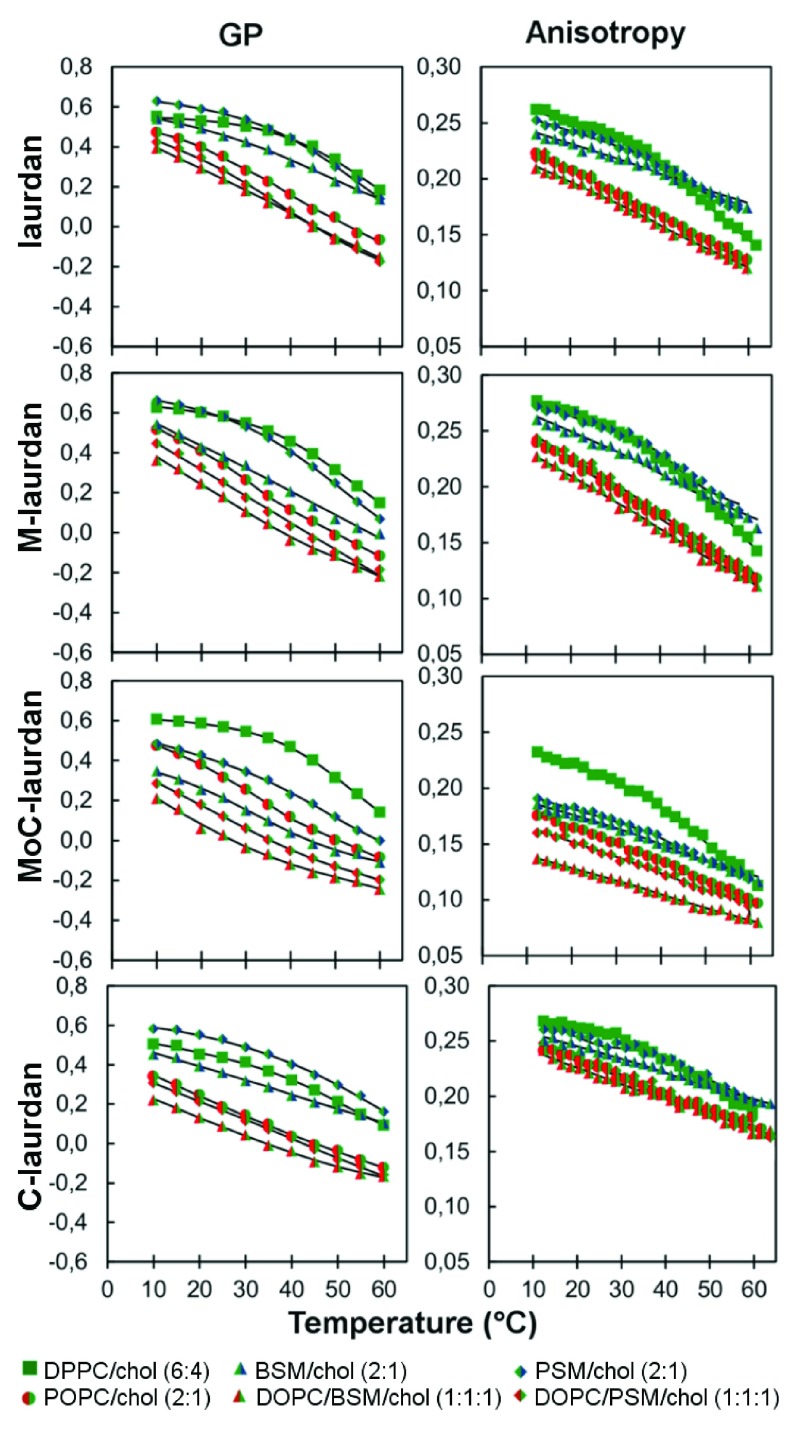
GP and anisotropy of laurdan, M-laurdan, MoC-laurdan and C-laurdan dyes in model membrane in Lo phase (green, blue/green and red/green color codes depicting liquid ordered and mixed phases).

Next, we performed experiments on bilayers formed with DPPC/cholesterol (6:4), a standard mix used to produce lipid bilayers in pure Lo phase ^48, 49^. As expected with this model system, no significant differences of GP or anisotropy characteristic of phase transitions was seen with any of the four as of temperature ( Figure 6).

We then prepared the very similar lipid mix as the one used by Kim *et al.* ^15^, i.e. DOPC/sphingomyelin/cholesterol (1:1:1), using either BSM or PSM, and confirmed their results showing intermediary values for GP, compared to pure So and Ld phases. Our interpretation, however, differs from theirs. Indeed, we believe that the resulting emission spectrum and the corresponding GP values could simply correspond to a juxtaposition of Ld phases, enriched in DOPC, and Lo phases, enriched in sphingomyelin and cholesterol, ( Figure 4 and Figure 6, Figure S5). Similar intermediary values were obtained in POPC/cholesterol (2:1) bilayers, in which Lo and Ld phases are known to coexist. In addition, intermediary values were also found for anisotropy. Furthermore, the interpretation that this mixture, DOPC/sphingomyelin/cholesterol (1:1:1) contains both Lo and Ld phases is in good agreement with previous publications ^37^.

In line with a hypothesis formulated by one of us ^50^ that solid docks could contribute to the formation of membrane microdomains ^51^, we were interested in measuring whether these probes could discriminate Lo from So phase when both phases were coexisting. For all four probes, coexistence of Lo and So phases in a sphingomyelin/cholesterol bilayer led to the disappearance of the phase transition observed in bilayers made of just sphingomyelin and analyzed using GP. Similar effects were observed by anisotropy. Altogether there thus does not seem to be one particular criterion for any of the four probes which would allow the clear-cut discrimination between So and Lo phases, especially in a biological membrane containing high levels of sphingolipids and cholesterol such as the plasma membrane of eukaryotic cells.

### Correlating GP/relaxation time to identify lipid phases

**Figure 7.**
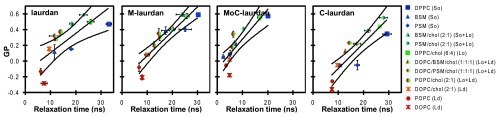
Correlation graphs between GP and relaxation time for laurdan, M-laurdan, MoC-laurdan and C-laurdan in various model membranes at room temperature (color codes reported from Figure 2– Figure 4). Experiments were performed twice and reported values are mean ± SD (unseen error bars are below the size of symbols). Superimposed lanes are linear regressions and 95% confidence bands. The correlation coefficient is the best for M-laurdan (0.83), good for C-laurdan (0.79) and MoC-laurdan (0.75) and moderate for laurdan (0.57). Beyond the coefficient of correlation, M-laurdan is the only probe for which pure sphingomyelins (BSM and PSM) are measured close to DPPC, reporting most accurately for bilayers in solid order phase at room temperature.

When a lipid bilayer goes from fluid to solid, both GP and relaxation time are expected to increase, but those two measurements actually reflect very different characteristics: whilst GP values are mostly influenced by the hydration of the environment, relaxation time reflects on the order parameter of the bilayer related to fluidity. When GP is plotted as a function of relaxation time for measurements made at room temperature on various model membranes ( Figure 7 and supporting information table S2), we find that M-laurdan is the only probe for which there is a reasonable correlation with the expected physical state of the bilayers, i.e. for which the bilayers made of just sphingomyelin (BSM or PSM), which are expected to be in solid state, do not give unexpectedly low values of GP and/or relaxation time. We perceive that this type of phenomenon could be due to the probes being excluded from the crystalline mesh of solid phases. The probes would consequently accumulate in the relatively disorganized environment corresponding to cracks and imperfections that form between tiles of truly organized lipids. In contrast with studies performed with F2N8, another polarity dye ^27^, our data suggest that, when measured with M-laurdan, hydration and fluidity are changing in a correlated manner in all the bilayer compositions tested, including pure sphingomyelins (BSM and PSM) measures falling close to those of DPPC. This result might come from an optimum location of the M-laurdan when inserted into the lipid bilayer as compared to laurdan, MoC-laurdan and C-laurdan, for which the carbonyl function present in sphingosine may interact with the polar heads of the probes, with possible formation of hydrogen bond between the probe and the hydroxyl residue or the amide linkage carried by the sphingosine ^20^. As a result, we think that the simultaneous measurement of GP and relaxation time with M-laurdan used as a probe will permit a true identification of the lipid phases.

### Labelling of live cells

Next, we compared the capacity of all four probes to label live cells. First, we used flow cytometry to determine the speed at which the probes became incorporated into COS7 cells, as well as the speed at which the probes came out of the cells after staining was completed ( Figure 3B-Right panel). As expected from what we had found with liposomes, staining with C-laurdan was much faster than with the other three probes, with maximum levels attained after just 20 minutes, while staining with the other probes was still increasing steadily after 40 minutes. The reverse was true, however, when it came to the stability of the staining, with the C-laurdan staining decreasing much more rapidly than for the other three probes. Of note, similar results were obtained when the probes were used to stain either HEK or Jurkat cells, and live gating with propidium iodide did not reveal any particular toxicity on any of the three cell types (unpublished data).

**Figure 8.**
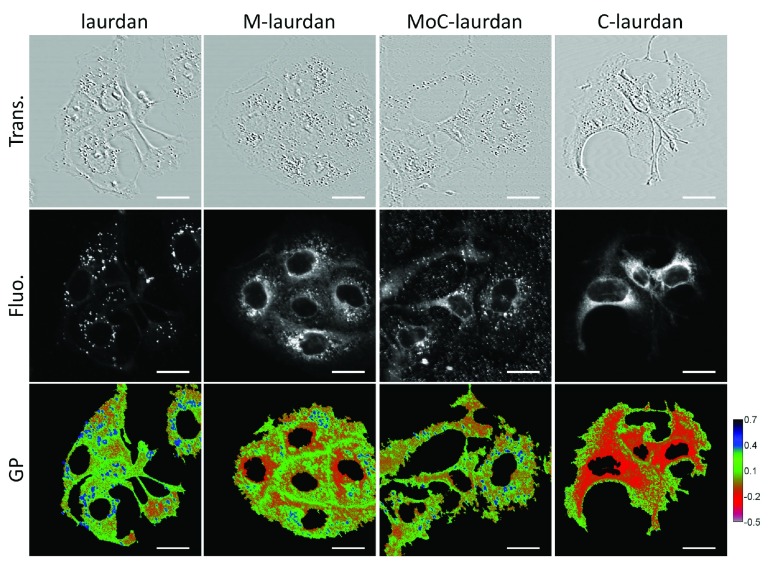
Two-photon imaging of COS7 cells labelled with the four different probes. Upper row: transmission (Trans.); middle row: total fluorescence (Fluo.) from 420 to 600 nm; bottom row: GP calculated with the values from 10 nm channels centered on 440 and 490 nm (scale bar, 20 µm).

**Supplemental Figure S6.**
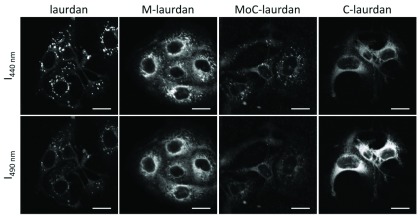
Fluorescence emission spectra recorded at 440 and 490 nm using the Zeiss LSM 710 confocal spectral mode (two-photon excitation set to 720 nm). The images are recorded in one pass on the spectra detector and used to calculate GP maps (scale bar, 20 µm).

To perform microscopy, we then used the probes to label COS7 cells adhered to glass coverslips, and labelled them as described in Materials and Methods. The coverslips were then placed at 37°C in the thermostatic chamber of a LSM 710 microscope, equipped for two-photon excitation. Using the spectral capacity of the microscope, the intensity images were recorded in one pass from 420 to 600 nm with 10 nm steps. Images recorded at 440 and 490 nm were used to calculate GP maps ( Figure S6). As shown in Figure 8, all four probes resulted in clear labelling of the cells, albeit with remarkably different patterns. With laurdan, the staining was predominantly present in intra-cytoplasmic vesicles. We suspect that those vesicles, which have high GP values, most probably belong to the endosome/lysosome compartment. Of note, a similar pattern of intra-cellular vesicles was also reported by Kim *et al.* ^15^ on A431 cells. Remarkably, those vesicles were absent from the staining obtained with C-laurdan, which showed a much more diffuse staining pattern, apart from a strong para-nuclear signal of low GP value which probably corresponds to the staining of the endoplasmic reticulum. Both the high GP vesicles and the low GP para-nuclear signal were present in the cells stained with M-laurdan, suggesting that this probe has a more ubiquitous distribution than laurdan or C-laurdan. Staining with MoC-laurdan resulted in patterns somewhat similar to those obtained with M-laurdan, but this probe also gave many undesirable foci on the coverslips outside of cells, which we suspect correspond to precipitated probe aggregates.

One feature which is common to all four probes is that the plasma membranes always show higher GP values than the intra-cellular compartments, which is in good agreement with the higher cholesterol content of the plasma membrane.

Data of M-laurdan characterization to explore order in lipid membranes Dataset 1: This dataset contains the raw data of spectroscopic properties in solvents. Measurement of spectroscopic properties, quantum yield (phi), lifetime (tau) and Generalized Polarization (GP, performed twice, reported values mean ± SD) of laurdan, M-laurdan, MoC-laurdan and C-laurdan in various solvents (chloroform, dichloromethane, acetone, acetonitrile, dimethylformamide, ethanol, methanol). Dataset 2: This dataset contains GP and anisotropy measurements for laurdan, M-laurdan, MoC-laurdan and C-laurdan recorded in various model membranes at room temperature (experiments were performed twice and data report for mean ± SD).
Dataset 3: Generalized polarization and relaxation time

## Conclusion

We have developed a simplified approach for the easy and efficient synthesis of 2-hydroxy-6-dodecanoyl naphthalene, the synthesis precursor of laurdan and C-laurdan as well as two new fluorophores, M-laurdan and MoC-laurdan. The measurements of the photophysical parameters performed on those four fluorophores solubilized in solvents showed that, for all of them, hydration has a higher impact than dielectric constant of the solvent. By combining GP and anisotropy measurements on the same model bilayers, we could simultaneously retrieve information related to the hydration level at the interface of the bilayer and to the rotational constraints of the probes. As already reported ^27^, we found that the presence of cholesterol results in reduced hydration of the bilayers, while the presence of sphingomyelin induces both an increase of GP, suggesting an increased hydration in the probes’ environment or extra H-bonding, and an increase of their apparent rotational mobility. This feature is particularly important given that sphingolipids comprise roughly 30% of the lipids in the plasma membranes of eukaryotes ^52^, and they play a critical role in the formation of lipid domains. In our data, M-laurdan, gave results which were best in agreement with the phases expected from the literature independently of the chemical nature of the lipids and of the fluorescence techniques in use. When used to label live cells, M-laurdan was also the probe which gave the most ubiquitous staining patterns, together with a good stability of the staining over time. M-laurdan thus appears as a promising tool for exploring lipid phases and order in biological membranes.

## Data availability

*figshare*: Data of M-laurdan characterization to explore order in lipid membranes, doi: http://dx.doi.org/10.6084/m9.figshare.1109901 ^53^

## Figures and Tables

**Supplemental Table S1.** Bibliographic references for the composition of the bilayers used to obtain de various lipid phases.

Composition and lipid phase at low temperature	Reference
DPPC (So) DPPC/chol 6:4 (Lo)	Vist, M. R.; Davis, J. H. Biochemistry 1990, 29, 451–464. Zhang, J.; Cao, H.; Jing, B.; Almeida, P. F.; Regen, S. L. Biophys. J. 2006, 91, 1402–1406.
PSM (So) PSM/chol 2:1 (So + Lo) POPC (Ld) POPC/chol 2:1 (Lo + Ld)	De Almeida, R. F. M.; Fedorov, A.; Prieto, M. Biophys. J. 2003, 85, 2406–2416.
DOPC (Ld) DOPC/chol 2:1 (Ld)	Davis, J. H.; Clair, J. J.; Juhasz, J. Biophys. J. 2009, 96, 521–539. Veatch, S. L.; Keller, S. L. Biophysical Journal 2003, 85, 3074–3083. M’Baye, G.; Mély, Y.; Duportail, G.; Klymchenko, A. S. Biophysical Journal 2008, 95, 1217–1225.
DOPC (Ld) DOPC/chol 2:1 (Ld) DOPC/PSM/chol 1:1:1 (Lo + Ld)	Nyholm, T. K. M.; Lindroos, D.; Westerlund, B.; Slotte, J. P. Langmuir 2011, 27, 8339–8350.
POPC (Ld)	Ionova, I. V.; Livshits, V. A.; Marsh, D. Biophysical Journal 2012, 102, 1856–1865.
BSM (So)	Meyer, H. W.; Bunjes, H.; Ulrich, A. S. Chem. Phys. Lipids 1999, 99, 111–123. Pokorny, A.; Yandek, L. E.; Elegbede, A. I.; Hinderliter, A.; Almeida, P. F. F. Biophysical Journal 2006, 91, 2184–2197.

**Supplemental Table S2.** Rotational relaxation time ρ (ns) of laurdan, M-laurdan, MoC-laurdan and C-laurdan at 20°C. Anisotropy values (r) were converted into rotational relaxation time (ρ) taking into account the average lifetime (τ) of the probes using the following equation: $\rho \;=\;\frac{3\tau }{\left(\frac{r_{0}}{r}\right)\;-\;1}$, [G. Weber, Adv. Prot. Res. 1953, 8, 415], where r _0_ is the intrinsic anisotropy (set to the theoretical maximal value of 0.4 for calculations).

Sample		laurdan				M-laurdan		
		r	τ (ns)	ρ (ns)		r	τ (ns)	ρ (ns)
**DPPC**		0.26	5.82	33.13		0.27	4.86	30.30
**PSM**		0.23	4.28	18.27		0.27	4.06	25.01
**BSM**		0.20	3.82	11.69		0.24	3.81	17.25
**DPPC/chol (6:4)**		0.25	5.41	25.87		0.26	4.45	25.70
**PSM/chol (2:1)**		0.24	5.34	22.96		0.26	4.36	23.68
**BSM/chol (2:1)**		0.23	4.61	18.27		0.24	4.29	20.20
**POPC/chol (2:1)**		0.21	4.35	14.01		0.22	3.98	14.71
**DOPC/BSM/chol (1:1:1)**		0.19	4.36	12.11		0.21	4.06	12.82
**DOPC/PSM/chol (1:1:1)**		0.19	4.30	11.41		0.23	3.85	15.05
**DOPC/chol (2:1)**		0.18	3.75	9.48		0.19	3.81	10.00
**DOPC**		0.18	3.04	7.40		0.18	3.33	8.01
**POPC**		0.16	3.19	6.09		0.17	3.16	6.81

Sample		MoC-laurdan				C-laurdan		
		r	τ (ns)	ρ (ns)		r	τ (ns)	ρ (ns)
**DPPC**		0.22	5.24	20.07		0.26	5.02	29.23
**PSM**		0.15	2.77	4.98		0.27	2.87	17.45
**BSM**		0.11	2.00	2.35		0.22	2.96	10.70
**DPPC/chol (6:4)**		0.22	4.73	16.75		0.26	4.69	26.31
**PSM/chol (2:1)**		0.18	4.03	10.34		0.26	5.05	27.84
**BSM/chol (2:1)**		0.17	3.26	6.88		0.25	4.44	21.46
**POPC/chol (2:1)**		0.16	3.21	6.27		0.23	3.53	14.46
**DOPC/BSM/chol (1:1:1)**		0.14	3.17	5.05		0.22	3.36	12.79
**DOPC/PSM/chol (1:1:1)**		0.15	3.16	5.94		0.24	3.72	17.38
**DOPC/chol (2:1)**		0.14	3.15	4.88		0.22	2.62	9.67
**DOPC**		0.13	3.17	4.83		0.21	2.13	7.42
**POPC**		0.11	3.03	3.51		0.21	2.26	7.58

## Synthesis of laurdan family products

2-hydroxy-6-dodecanoylnaphthalene: Condensation of Naphtol-2 with Lauroyl chloride in presence of tri-ethanol-amine gave naphtyl-laurate with a yield of almost 100% and the crude product was analyzed by thin layer chromatography and ^1^H NMR (500 MHz, CDCl _3_). δ = 7.90 (dd, J=1.0, 3.0 Hz, 1 H), 7.85 (dd, J=9.0, 3.0 Hz, 2 H), 7.60 (d, J=1.0 Hz, 1 H), 7.52 (qd, J=9.0 Hz, 2 H), 7.35 (dd, J=9.0, 3.0 Hz, 1 H), 2.65 (t, 2 H), 1.7 (quin, J=7.5 Hz, 2 H), 1.35 (m, 16 H), 0.88 (t, J=7.5 Hz, 3 H). Then, a Fries rearrangement was carried out with anhydrous methane sulfonic acid as solvent and reactant (Journal of Molecular Catalysis A : chemical 182–183 (2002) p. 137–141 “Fries rearrangement in methane sulfonic acid an environmental friendly acid” A. Commarieu, W Hoelderich, J. A; Laffitte, M-P Dupont). The naphtyl-laurate obtained previously (0.073 mol) and anhydrous acid methane sulfonic (1.98 mol, 27 equiv.) were mixed in a 250 ml three-necked flask, equipped with a cooler and stirrer. The system was flushed with nitrogen and heated at 90°C for 1 h. The medium turned red. After cooling at room temperature, 125 g dichloromethane was added and the mixture was then poured in a container placed on crushed ice. After stirring for 1 h, the organic phase was separated and the aqueous phase extracted with 200 g of dichloromethane. The organic phases were combined and dried on magnesium sulfate. Dichloromethane was evaporated, resulting in a crude product which was analyzed by thin layer chromatography and ^1^H NMR. (500 MHz, CDCl _3_): δ = 8.42 (s, 1 H), 8.03 (dd, J=9.0, 3.0 Hz, 2 H), 7.90 (dd, J=1.0 Hz, 1 H), 7.72 (d, J=9.0 Hz, 1 H), 7.20 (s, 2 H), 6.35 (dd, J=9.0, 3.0 Hz, 1 H), 3.1 (t, 2 H), 1.7 (quin, J=7.5 Hz, 2 H), 1.39 (m, 16 H), 0.91 (t, J=7.5 Hz, 3 H). This analysis revealed that the rearrangement of naphtyl-laurate in 2-hydroxy-6-dodecanoy-napahthalene was also carried out with a yield of almost 100%.

Laurdan: di MeNH•HCl (11.0 g, 0.13 mol) was added to a mixture of 2-hydroxy-6-dodecanoylnaphthalene (8.0 g, 26 mmol), Na _2_S _2_O _5_ (12 g, 61 mmol), NaOH (5.2 g, 0.13 mol), and H _2_O (100 ml) in a pressure tube and the mixture was stirred at 140°C for 48 h. The product was collected by filtration, washed with water and purified by crystallization from CH _2_Cl _2_/EtOH; yield 5.8 g (69%); ^1^H NMR (500 MHz, CDCl _3_): δ = 8.35 (s, 1 H), 7.96 (dd, J=9.1, 3.0 Hz, 1 H), 7.73 (d, J=9.1 Hz, 1 H), 7.68 (d, J=9.1 Hz, 1 H), 6.93 (dd, J=9.1, 3.0 Hz, 1 H), 6.77 (s, 1 H), 3.03 (t, J=7.5 Hz, 2 H), 2.97 (s, 6 H), 1.77 (quin, J=7.5 Hz, 2 H), 1.26 (m, 16 H), 0.88 (t, J=7.5 Hz, 3 H).

M-laurdan: MeNH _2_•HCl (9.0 g, 0.13 mol) was added to a mixture of 2-hydroxy-6-dodecanoylnaphthalene (8.0 g, 26 mmol), Na _2_S _2_O _5_ (12 g, 61 mmol), NaOH (5.2 g, 0.13 mol), and H _2_O (100 ml) in a pressure tube and the mixture was stirred at 140°C for 48 h. The product was collected by filtration, washed with water and purified by crystallization from CH _2_Cl _2_/EtOH; yield 5.6 g (67%); ^1^H NMR (500 MHz, CDCl _3_): δ = 8.33 (s, 1 H), 7.95 (dd, J=9.1, 3.0 Hz, 1 H), 7.74 (d, J=9.1 Hz, 1 H), 7.66 (d, J=9.1 Hz, 1 H), 7.29 (s, 1 H), 6.91 (dd, J=9.1, 3.0 Hz, 1 H), 6.77 (s, 1 H), 3.03 (t, J=7.5 Hz, 2 H), 2.97 (s, 3H), 1.77 (quin, J=7.5 Hz, 2 H), 1.26 (m, 16 H), 0.88 (t, J=7.5 Hz, 3 H).

C-laurdan: A mixture of 6-dodecanoyl-2-(methylamino) naphthalene (3.0 g, 8.8 mmol), methyl bromoacetate (2.0 g, 13 mmol), Na _2_HPO _4_ (1.9 g, 13 mmol) and NaI (0.5 g, 3.5 mmol) in MeCN (50 ml) was refluxed under N _2_ for 18 h. The product was extracted with ethyl acetate, washed with brine and purified by crystallization from EtOH to obtain a light yellow powder; yield 2.5 g (70%); ^1^H NMR (500 MHz, CDCl _3_): δ = 8.35 (s, 1 H), 7.93 (dd, J=9.0, 3.0 Hz, 1 H), 7.82 (d, J=9.0 Hz, 1 H), 7.68 (d, J=9.0 Hz, 1 H), 7.08 (dd, J=9.0, 3.0 Hz, 1 H), 6.83 (s, 1 H), 4.23 (s, 2 H), 3.72 (s, 3H), 3.24 (s, 3 H), 3.04 (t, J=7.5 Hz, 2 H), 1.77 (quin, J=7.5 Hz, 2 H), 1.28 (m, 16 H), 0.89 (t, J=7.5 Hz, 3 H); A mixture of this intermediate (2.0 g, 4.9 mmol) and KOH (0.70 g, 12 mmol) in EtOH (50 ml) was stirred for 5 h. The resultant solution was diluted with ice-water (100 ml) and concentrated HCl (aq) was added slowly at <5°C until pH 3 was reached. The resulting precipitate was collected, washed with distilled water and purified by crystallization from chloroform/petroleum ether; yield 1.5 g (79%); ^1^H NMR (500 MHz, CDCl _3_): δ = 12.77 (br s, 1 H), 8.46 (s, 1 H), 7.91 (d, J=9.0 Hz, 1 H), 7.82 (d, J=9.0 Hz, 1 H), 7.67 (d, J=9.0 Hz, 1 H), 7.21 (d, J=9.0 Hz, 1 H), 6.95 (s, 1 H), 4.26 (s, 2 H), 3.12 (s, 3 H), 3.05 (t, J=7.5 Hz, 2 H), 1.68 (quin, J=7.5 Hz, 2 H), 1.29 (m, 16 H), 0.85 (t, J=7.5 Hz, 3 H).
