# Supplementary material for: Characterization of M-laurdan, a versatile probe to explore order in lipid membranes
Source: F1000Res. 2014 Nov 19;3:172. Originally published 2014 Jul 25. [Version 2] doi: 10.12688/f1000research.4805.2 (PMC4243762; doi:10.12688/f1000research.4805.2)
Supplement: Peer review history

# Peer review: Referee response for version 2

**DOI:** 10.5256/f1000research.6071.r6742

**Published:** 2014-11-21

**Authors:** John D Bell

**Affiliations:** 1 The College of Life Sciences, Brigham Young University, Provo, USA

The latest revisions have improved the paper.

I have read this submission. I believe that I have an appropriate level of expertise to confirm that it is of an acceptable scientific standard.

# Peer review: Referee response for version 1

**DOI:** 10.5256/f1000research.5129.r5878

**Published:** 2014-09-19

**Authors:** John D Bell

**Affiliations:** 1 The College of Life Sciences, Brigham Young University, Provo, USA

This paper describes the synthesis and characterization of two new forms of the well-known membrane fluorescent probe, Laurdan. The paper is interesting and generally well written. The work is of interest to researchers in the field.

I have read the comments of the other reviewers of the paper and agree with their concerns, adding my endorsement of the need to respond to their points. In particular, I also think that Fig. 7 is not a good choice for arguing the superiority of M-Laurdan. As pointed out by Dr. Cherezov, one cannot judge the strength of the correlations without quantitative information. My guess, looking at the figure, though, is that the differences are small. It would, though, be better to compare GP to the rotational relaxation time as suggested by Dr. Duportail since GP and anisotropy are mutually convoluted with excited-state life time.

More importantly, it is not clear to me why a strong correlation between the two parameters (GP and anisotropy) is necessarily ideal. Since the two represent different things (polarity of local environment vs. probe wobble (after correcting for lifetime)), isn't there value in exploring conditions under which they may report disparate results? Frankly, I am more excited about the richness of information that could come from comparisons among probes that interact differently with membrane lipids (as revealed by Fig. 5) than by choosing a single probe based on the concept that two different measurements with the probe are best if they are redundant. I would therefore recommend that the paper be steered more in the direction of pointing out the types of information each probe can provide rather than trying to identify one as the optimum.

I have read this submission. I believe that I have an appropriate level of expertise to confirm that it is of an acceptable scientific standard, however I have significant reservations, as outlined above.

# Peer review: Referee response for version 1

**DOI:** 10.5256/f1000research.5129.r5962

**Published:** 2014-09-02

**Authors:** Guy Duportail

**Affiliations:** 1 Laboratoire de Biophotonique et Pharmacologie, University of Strasbourg, Strasbourg, France

This work from Mazeres *et al.* deals with a family of membrane fluorescent probes of the well-known Laurdan family, more precisely Laurdan itself (as “mother” probe), C-Laurdan which was already introduced and studied by Kim *et al.* ( *Chembiochem*, 2007, 8, 553) and two new derivatives, so-called M-Laurdan and MoC-Laurdan. All these derivatives were synthesized by the authors through simplified chemical steps. The paper mainly presents an extensive characterization of the fluorescence properties of the probes in organic solvents of different polarities and dielectric constants, and in lipid vesicles of varying compositions, focusing on the fluorescence response versus lipid phases, either solid, liquid ordered (raft) or liquid disordered phases. Some preliminary results are also presented concerning live cells labelling. Visualization of lipid domains still remains a challenge, especially in cellular membranes. It is clear that the search and studies of new environment-sensitive membrane probes constitute an important issue in the development of raft imaging tools for cellular applications. In this context, the present work is an interesting contribution to progress in this challenge. However, either to improve the present contribution or to get more clear and precise information in a following work, we have to underline some critical points:

 

- Materials and Methods: Large Unilamellar Vesicles (LUVs) were prepared by sonication, as claimed by the authors. Such a method is probably not giving LUVs, but rather, depending on the delivered power, either Small Unilamellar Vesicles (SUVs), or an homogenized distribution of Multilamellar vesicles (MLVs) or Oligolamellar vesicles (OLVs). According to the sizes here presented (150 to 350 nm), I guess we are in presence of the latter case. This is not so important in the present work, since the fluorescence parameters presently collected are responding more or less identically whatever the type of lipid vesicles, but for example if it will be question to study the precise localization of a probe within a lipid bilayer, LUVs must be used. Generally the best procedure to get them is either extrusion or Kachel’s method ( *BBA,*1998,1374, 63).
- How to interpret fluorescence anisotropy in terms of order parameter? The authors are fully aware that the fluorescence anisotropy ( *r *) as a measure of membrane order must take into account the fluorescence lifetime ( τ ) of the probe in the medium. They present a lot of data for both parameters, but in different tables, and the corresponding discussion is hard to unravel. Why not to present the rotational relaxation time parameter ρ which integrates *r *and τ and is directly correlated with the order parameter through the Perrin-Weber equation [( *r _0_/r)* – 1] = 3 τ/ρ (cf. G. Weber, *Adv.Prot. Res. *1953, 8, 415 *). *We are presently in an ideal case to use this average parameter, since we are comparing probes presenting the same fluorophore, and consequently more or less the same molecular volume.
- For further works with these probes, it should be very interesting to proceed to parallax fluorescence quenching experiments in order to know the precise location of these probes (depth of the fluorophore in the lipid bilayer). This would be of great help for the interpretation of several data. In this case, experiments must be performed by using identical and well characterized LUVs.

 

Minor comments and suggestions:

 

- Introduction, page 4. It is not true that plasma membranes are mainly composed of PC, SM and Chol. It is true only for the external leaflet. The inner leaflet contains significant amount of PS and to a lesser extent PE.
- Fig.1: data are obtained in chloroform. Better to indicate in the title than twice in footnotes.
- Labelling of LUVs, page 6. I do not catch what are “steps of dessication and rehydration”? Is it steps of freezing and defreezing?
- As compared to the three other probes, C-Laurdan is at least partially ionized, due to its carboxylic group. This explains its water solubility, why its incorporation in lipid vesicles or cell membranes is very rapid, and probably also some of its peculiar data in protic solvents. This should be mentioned.
- To compare data obtained with DPPC and SMs (either BSM or PSM), we should not forget that SMs are not single molecular species as DPPC, so that it is not surprising that, for example, a phase transition followed by fluorescence anisotropy cannot be so clear-cut with SMs as with DPPC.
- In their conclusion, the authors are presenting the ubiquitous staining patterns of M-Laurdan in cells as an advantage. It is not so evident as very often a need exists for a selective labelling (for example plasma membrane, or mitochondrial membrane or endosomal membrane).

I have read this submission. I believe that I have an appropriate level of expertise to confirm that it is of an acceptable scientific standard.

# Peer review: Referee response for version 1

**DOI:** 10.5256/f1000research.5129.r5595

**Published:** 2014-08-26

**Authors:** Vadim Cherezov

**Affiliations:** 1 Department of Integrative Structural and Computational Biology, Scripps Research Institute, La Jolla, CA, USA

This article describes an improved procedure for the synthesis of two fluorescent probes commonly used to image lipid rafts: laurdan and C-laurdan, as well as two new probes: M-laurdan and MoC-laurdan. All four probes are extensively characterized in different solvents and membranes with different lipid compositions in the temperature range from 10 to 60 °C.

 Based on presented data, the authors conclude that the new probe, M-laurdan, is the best of four for discrimination of lipid membrane phases independently of the chemical nature of lipids. This conclusion appears to stem mainly from the correlation plots between GP and anisotropy shown in Fig. 7. The authors describe correlations as the strongest for M-laurdan, good for laurdan and MoC-laurdan, and moderate for C-laurdan. Just from looking at Fig. 7, however, it is not obvious that the correlation is the strongest for M-laurdan. It would help if the authors will provide a quantitative measure of correlation, such as a correlation coefficient or another similar parameter.

The authors also state that M-laurdan makes the best staining patterns in live cells. Based on Figure 8, the difference between laurdan, M-laurdan and MoC-laurdan is not very obvious. It would be great if either a better example or better explanations are provided.

Other comments and suggestions are listed below:

1. Some measurements were taken two times and the mean values and standard deviations were provided, e.g. Fig. 2, Fig. 7. Many other measurements seem to be only performed once, for example,  Figures 3, 4, 6, S1, S2, S4, Tables 2, 3). I would suggest performing at least 3-4 measurements and report standard deviations for all measured values.
2. Results and Discussion - Insertion of the fluorophores into LUVs:  It is stated that no detectable difference is found comparing two methods of labeling referring to Figure 4. However, Figure 4 only shows data for the DMSO loading method and not for direct mixing. Also, it would be great to see the same data for M-laurdan and MoC-laurdan.
3. The section title “Probing model bilayers in pure So states” is misleading, as this section describes mostly transition from So to Ld phase. The same applies to Fig.5, where it says that blue symbols depict So phase.
4.  Results and Discussion - Probing model bilayers in Lo states: In the second paragraph Kim *et al.* should have a reference associated with it.

I have read this submission. I believe that I have an appropriate level of expertise to confirm that it is of an acceptable scientific standard, however I have significant reservations, as outlined above.
